# Prevention of new-onset atrial fibrillation in elderly patients undergoing anatomic pulmonary resection by infusion of magnesium sulfate: protocol for a randomized controlled trial

**DOI:** 10.3389/fcvm.2023.1171713

**Published:** 2023-11-17

**Authors:** Shuqing Jin, Long An, Linsong Chen, Huqing Liu, Hongfei Chen, Xin Lv

**Affiliations:** ^1^ Department of Anesthesiology, Shanghai Pulmonary Hospital, School of Medicine, Tongji University, Shanghai, China; ^2^ Department of Anesthesiology, Kashgar Regional Second People’s Hospital, Xinjiang, China; ^3^ Department of Anesthesiology, Tongren Hospital, Shanghai Jiao Tong University School of Medicine, Shanghai, China

**Keywords:** atrial fibrillation, anatomic pulmonary resection, older patients, magnesium sulfate, randomized controlled trial

## Abstract

Atrial fibrillation (AF) is the most commonly sustained arrhythmia after pulmonary resection, which has been shown to predict higher hospital morbidity and mortality. The lack of strong evidence-based medical evidence makes doctors have very few options for medications to prevent new-onset AF following thoracic surgery. Magnesium can prevent perioperative AF in patients undergoing cardiac surgery. However, this has not yet been fully studied in patients undergoing non-cardiac thoracic surgery, which is the aim of this study. This is a single-center, prospective, double-blind, randomized controlled trial. In total, 838 eligible patients were randomly assigned to one of two study groups, namely, the control group or the magnesium group. The patients in the magnesium group preoperatively received 80 mg magnesium sulfate/kg ideal weight in 100 ml normal saline 30 min. The control group received the same volumes of normal saline simultaneously. The primary outcome is the incidence of new-onset AF intra-operative and on the first, second, and third postoperative days. The secondary outcomes are bradycardia, hypertension, hypotension, and flushing. The occurrence of stroke or any other type of arrhythmia is also recorded. Postoperative respiratory suppression and gastrointestinal discomfort, intensive care unit stays and total duration of hospital stays, in-hospital mortality, and 3-month all-cause mortality are also recorded as important outcomes. This study aims to prospectively evaluate the prophylactic effects of magnesium sulfate against AF compared with a placebo control group during and following anatomic pulmonary resection. The results may provide reliable evidence for the prophylactic value of magnesium against AF in patients with lung cancer. The trial was approved by the Clinical Research Ethics Committee of Shanghai Pulmonary Hospital and has been registered at **Chinese Clinical Trial Registry**: www.chictr.org.cn, identifier: ChiCTR2300068046.

## Introduction

The incidence rate of atrial fibrillation (AF) ranges from 6% to 40% after non-cardiac thoracic surgery, which is known to be the most commonly sustained arrhythmia (1–7). Following thoracic surgery, most AFs are newly diagnosed (8). New-onset AF refers to the first occurrence of AF in an individual with no prior history of diagnosed AF. Perioperative AF is a high-risk factor for short-/long-term stroke after thoracic surgery. It is also strongly associated with higher hospital morbidity and mortality rates in both short term and long term after general thoracic surgery, longer intensive care unit (ICU) stays and hospital stays, and consequent higher resource utilization (6, 8–16).

Older patients are more likely to develop new-onset AF during or after surgery. Older age was consistently reported as an independent preoperative risk factor most strongly associated with AF (17). Aside from preoperative risk factors such as advanced age, a history of peripheral vascular disease, arrhythmias, and heart failure, male sex, resection of lobectomy, pneumonectomy, mediastinal tumor, and esophagectomy are thought to be independent predictors of the occurrence of AF at multivariate analysis (16, 18, 19).

To prevent the high incidence rate of AF, studies should focus on the effectiveness of pharmacologic interventions after thoracic surgery. While most data are from cardiac surgery, it should be noted that data regarding non-cardiac surgery in the field of lung cancer remain very limited (16).

Not many medications can be used to prevent AF. To date, amiodarone seems to be the most efficient prophylactic for AF after thoracic surgery. Amiodarone can control the ventricular rate and maintain the sinus rhythm (16). While it was reported that amiodarone could induce pulmonary toxicity in lung transplantation or right lung resection surgery, this medication is not widely used (20). In fact, we do not routinely use any drug to prevent AF after surgery because of the lack of strong evidence-based medical support. In our hospital, the approximately 20,000 non-cardiac thoracic surgeries every year and the high incidence of new-onset AF compel us to conduct high-quality clinical studies to find better preventive measures to reduce the incidence of perioperative AF in elderly patients.

As one of the most physiological ions, magnesium can stabilize membrane function to preserve cardiac rhythm (21). Using magnesium sulfate to prevent tachyarrhythmias has a long history. Magnesium supplementation is strongly recommended to effectively prevent postoperative AF in patients undergoing cardiac surgery (22). Only two prospective studies explored the role of magnesium sulfate in preventing AF in patients who underwent non-cardiac thoracic surgery. A comparative study by Khalil et al. published in 2013 evaluated the prophylactic effect of amiodarone and magnesium sulfate as antiarrhythmic agents against AF, showing that both amiodarone and magnesium sulfate can effectively prevent the incidence of AF during the intra- and postoperative periods following pulmonary resection surgery compared with the control group (23). The control patients in their research were retrospectively studied, and the average age of the patients in the magnesium sulfate group and the control group was 64.2 ± 3.8 and 63.7 ± 3.1 years, respectively. Since it is well proved that age is the most important factor in the occurrence of AF, our study only included older patients. Another prospective study focusing on patients undergoing non-cardiac thoracic surgery evaluated the possible efficacy of magnesium in preventing AF. It concluded that infusing MgSO_4_ can effectively and safely reduce the incidence of atrial tachyarrhythmias (2). In this study, digoxin was given to parts of patients in the control group, influencing the results. Another limitation was that they did not stratify different operations, as it is known that the type of operation affects the incidence of AF.

It should be noted that there is a dearth of data indicating the prophylactic medication for AF, which can effectively improve outcomes for non-cardiac thoracic surgery. In addition, many recommendations are extrapolated from the cardiac surgery arena. In this study, we prospectively evaluated the prophylactic effects of magnesium sulfate against new-onset AF compared with a placebo control group during and following anatomic pulmonary resection.

## Materials and methods

### Ethical approval

The study was conducted according to the principles of the *Declaration of Helsinki* and national regulations. The Ethics Committee of Shanghai Pulmonary Hospital approved this study protocol (version 1.3, 10 December 2022). To participate in this study before intervention, the patients/participants provided their written informed consent.

### Study design

**Figure 1:**
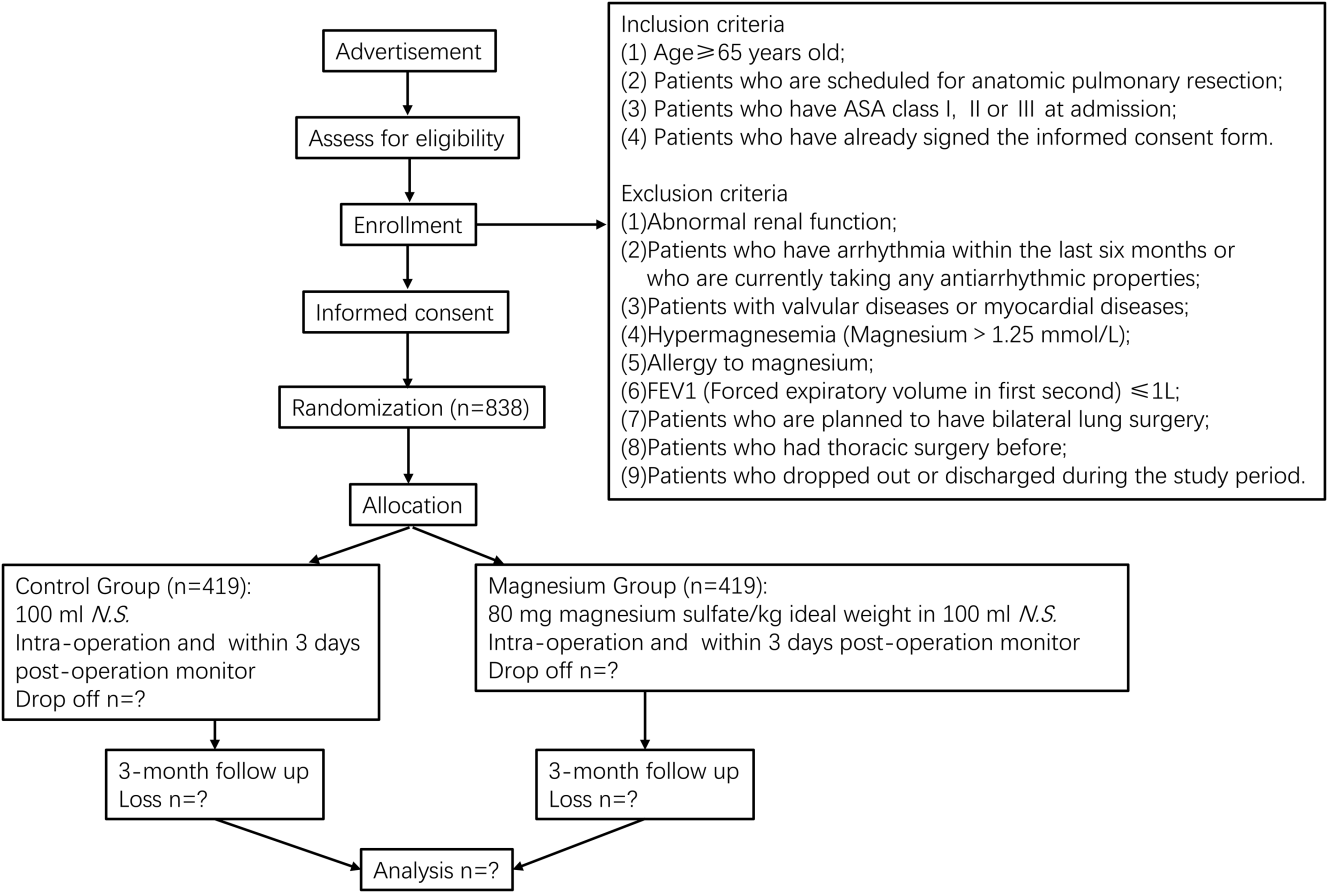
Flowchart of the study design. *N.S*., normal saline.

This is a single-center, prospective, double-blind, randomized controlled trial, designed according to the Standard Protocol Items: Recommendations for Intervention Trials (SPIRIT) 2013 statement (24). This trial has been registered at the Chinese Clinical Trial Registry (www.chictr.org.cn, identifier: ChiCTR2300068046). Figure 1 outlines the flow diagram of the protocol. Table 1 outlines the demonstration of enrollment, interventions, and assessment.

**Table 1:** Details of the planned schedule.

	Baseline	Follow-up period		
	Day 0 (preoperative)	Day 1 (during operation)	Days 1–3 (postoperative, during hospitalization)	Days 3–90 (postdischarge)
Included or excluded	✗			
Signed informed consent forms	✗			
Medical history^a^	✗			
Physical examination^b^	✗			
ECG	✗	✗	✗	
Lung function	✗			
Blood routine test	✗			
Biochemical test^c^	✗	✗	✗	
CHA_2_DS_2_-VASc score	✗			
Atrial fibrillation	✗	✗	✗	✗
Bradycardia	✗	✗	✗	✗
Hypotension	✗	✗	✗	
Hypertension	✗	✗	✗	
Flushing		✗	✗	
Any other type of arrhythmia	✗	✗	✗	✗
Respiration depression			✗	
Gastrointestinal discomfort			✗	
ICU stays			✗	
Total duration of hospital stays			✗	
All-cause mortality			✗	✗
Stroke		✗	✗	✗

^a^ Medical history includes the patient's past medical history, history of disease treatment, history of drug allergies, history of surgical trauma, history of blood transfusion, and especially cardiovascular diseases and their treatment history.

^b^ Physical examinations include blood pressure, heart rate, pulse, body temperature, height, and body mass.

^c^ Biochemical test parameters include electrolyte levels: measuring levels of sodium, potassium, and magnesium; liver function tests: evaluating liver enzymes and bilirubin; and kidney function tests: assessing levels of creatinine and blood urea nitrogen (BUN).

### Participants

The participants were recruited from Shanghai Pulmonary Hospital. Patients aged 65 years or older hospitalized for anatomic pulmonary resection were asked for informed consent first and then screened according to the inclusion and exclusion criteria.

### Inclusion criteria

(1) Age ≥65 years;
(2) Patients who are scheduled for anatomic pulmonary resection;
(3) Patients who have American Society of Anesthesiologists (ASA) class I, Ⅱ, or Ⅲ at admission;
(4) Patients who have already signed the informed consent form.

### Exclusion criteria

(1) Abnormal renal function;
(2) Patients who have arrhythmia within the last 6 months or are currently taking any antiarrhythmic properties;
(3) Patients with valvular diseases or myocardial diseases;
(4) Hypermagnesemia (magnesium >1.25 mmol/L);
(5) Allergy to magnesium;
(6) FEV1 (forced expiratory volume in first second) ≤1 L;
(7) Patients who are scheduled to have bilateral lung surgery;
(8) Patients who had thoracic surgery before;
(9) Patients who dropped out or were discharged during the study period.

### Sample size

In accordance with the incidence of AF from the previous study, the postoperative AF rates were 12.5% and 20.5%, respectively, in the magnesium and control groups (23). The type-Ⅰ error rate (α) was set at 0.05, and the type-Ⅱ error rate was set at 0.2. The sample size was calculated using software PASS 21: Proportions-Two Independence Proportions-Tests for Two Proportions. The sample size of each group was estimated to be 335. Considering a 20% drop-off rate, the sample size should be 419 in each group. Finally, a total of 838 subjects were recruited in this prospective study.

### Randomization, allocation concealment, and blinding

After informed consent was received, participants were randomized to the magnesium and control groups at a 1:1 ratio. An independent, blinded statistician generated a random allocation sequence using a research randomizer (http://www.randomizer.org). Randomization was based on preallocated block sizes and stratified by gender; the block size was set to four. Sequentially numbered envelopes, which were opaque and sealed, were used to achieve allocation concealment. The anesthesiologists, participants, and statisticians were blinded to the allocation, which was maintained until the completion of the final analyses. A nurse was in charge of the drug preparation and infusion. The anesthesiologists and surgeons involved in the assessment did not allocate the subjects.

### Intervention

A clinical evaluation was conducted before surgery. According to our institutional protocol, the patients received standard perioperative care. A central line was started (usually the right internal jugular vein) for all patients after monitoring pulse oximetry (SpO_2_), non-invasive blood pressure (BP), and a 5-lead electrocardiogram (ECG) scan. The patient's serum magnesium levels were well monitored before the surgery, 30 min after the start of the surgery, and on the first day after the surgery.

The induction of general anesthesia was performed using standard techniques, including dezocine 5 mg, midazolam 5 mg, fentanyl 0.5–1 μg/kg, propofol 1–2 mg/kg, and rocuronium 1 mg/kg. One-lung ventilation was achieved by inserting a visible double-lumen tube.

Anesthesia was maintained via continuous intravenous boluses of 4–12 mg/kg/h propofol and 5–10 μg/kg/h remifentanil with a 60% oxygen/air mixture. A single shot of 20 mg rocuronium was given when required. Serial blood gas analysis, urine output, and capnography were used to monitor acid–base balance, electrolytes, oxygenation, and ventilation. The tidal volume and respiratory frequency were set at 5–6 ml/kg and 12–14 breaths/min, respectively. In contrast, the tidal volume was adjusted to maintain the end-tidal carbon dioxide tension (PETCO_2_) at 40 ± 5 mmHg.

Before the start of the operation, patients were randomly allocated to one of the two groups consisting of 419 patients each, using sealed envelopes. The patients in the magnesium group received 80 mg magnesium sulfate/kg ideal weight in 100 ml normal saline 30 min before surgery, while the control group received an equivalent volume of normal saline for the same duration.

After extubation, all patients were transferred to the postanesthesia care unit and monitored for 1 hour. During the hospital stay, the patients were monitored in the surgical ward until discharge. The heart rate, blood pressure, ECG scan, and other routine hemodynamic monitors were used to detect AF.

Continuous 48-h ECG telemetry and oxygen saturation monitoring was performed on patients during the first 2 days after surgery. An anesthesiologist or surgeon diagnosed AF according to the definition in the 2014 Annual Meeting of the American Association for Thoracic Surgery (AATS) guidelines for the prevention and management of perioperative AF and flutter for thoracic surgical procedures (16). The presence of characteristic ECG features was recorded if AF lasted for at least 30 s (15). The anesthetic, fluid volume, and infusion speed were adjusted to maintain stable hemodynamics.

### Outcomes

The primary outcome is the incidence of new-onset AF intra-operatively and on the first, second, and third postoperative days. Bradycardia (<40/min), hypertension (>20% baseline), hypotension (<20% baseline), and flushing were recorded as secondary outcomes. The occurrence of stroke or any other type of arrhythmia is also recorded during hospitalization. Postoperative respiratory suppression and gastrointestinal discomfort, ICU stays and total durations of hospital stays, in-hospital mortality, and 3-month all-cause mortality were also recorded as important outcomes. Within 3 months after discharge, an event was recorded if a patient experienced a stroke or visited any medical institution for arrhythmia.

### Data management

Based on the case-reported forms (CRFs), an EpiData database was established. Two independent researchers timely and accurately filled in the CRFs and databases back-to-back. The study supervisor should supervise the conduct of the trial, which was performed every 3 months. If necessary, the chief investigator will make the datasets available upon reasonable request.

### Data analysis

All statistical analyses were performed using Statistical Product and Service Solutions (SPSS) 26.0 (IBM SPSS Inc., Chicago, IL, USA) statistical software. *P *< 0.05 (two-sided) was considered statistically significant. The data were collected using the *χ*^2^ or Fisher's exact test as appropriate and expressed as frequency with percentage (*N*, %) for categorical variables. Normally distributed quantitative data were evaluated using independent Student's *t*-test and expressed as the mean ± standard deviation (SD). Non-normally distributed quantitative data were determined using the Wilcoxon signed-rank test or Mann–Whitney *U*-test and expressed as median with interquartile range (IQR).

## Discussion

Atrial tachyarrhythmias have a particularly high incidence rate in thoracic surgery; most arrhythmias are AF. More than 75% of postoperative AFs occurred within the third day of the operation (2). Approximately 33% of AFs occurred on the second postoperative day (16, 25, 26).

Data on pharmacological prophylactic against new-onset AF in patients undergoing general thoracic surgery are mainly dependent on cardiac surgery. In addition, despite the high incidence rate of perioperative AF, drugs are not widely used to prevent AF peri-operation in non-cardiac thoracic surgery.

White and Hartzell proposed that altering Mg^2+^ could profoundly affect cardiac physiology. Autonomic control of the heart is dependent upon Mg^2+^ in numerous ways: binding of neurotransmitters to their receptors, coupling of receptors to adenylate cyclase, activation of G-proteins and adenylate cyclase, activation of proteins by Mg^2+^-dependent phosphotransferases, rectification of various types of K^+^ channels, activity of phosphorylated Ca^2+^ channels, voltage-dependent inactivation of Ca^2+^ channels, and optimal activity of mechanisms which maintain [Ca^2+^]i at resting levels (27). Administrating intravenous magnesium to patients with normal serum electrolyte levels caused a significant decrease in sinus node and atrioventricular (AV) node functions. Therefore, magnesium might be useful in treating arrhythmias whose mechanism depends on the participation of the sinoatrial or AV node such as supraventricular arrhythmias (28, 29).

The perioperative parenteral infusion of magnesium has been proven safe in patients with normal renal function, even at high dosages (30). Many studies have investigated the efficacy of magnesium in preventing AF after cardiac surgery. A review including 21 studies and a total of 2,988 participants demonstrated a significant reduction of postoperative AF and supraventricular tachycardia after magnesium treatment without significant side effects (31). By now, only two controlled studies evaluated the prophylactic effects of magnesium during and following non-cardiac thoracic surgeries (2, 23). One study published in 1996 by Terzi et al. reported that MgSO_4_ infusion was effective and safe for the reduction of postthoracotomy atrial tachyarrhythmias. They excluded video-assisted thoracic surgeries (VATS), while most thoracic surgeries in our hospital are VATS. Another limitation was that, in cases of pneumonectomy or intrapericardial procedure, the older patients (over 70 years old) in the control group received digoxin (0.5 mg) after surgery starting on the day of the operation. However, large randomized clinical trials have clearly proved and demonstrated that taking digoxin in advance does not prevent perioperative AF. In fact, in patients undergoing all types of thoracic surgery, it may increase the incidence of AF (16, 32–34). In addition, they also did not stratify different types of operations, as we know that lobectomy and esophageal resections are risk factors for perioperative AF, and it may result in bias. The other comparative study was unblinded, and the control group patients were studied retrospectively. As the author said, conducting a placebo-controlled, double-blinded prospective study is better.

This is a study protocol for a single-center, randomized, placebo-controlled, double-blind prospective clinical trial. The work aims to study the efficacy and safety of magnesium sulfate in preventing AF in elderly patients undergoing non-cardiac thoracic surgeries. The results will provide reliable evidence for the prophylactic value of magnesium in lung cancer patients.

## Ethics statement

The studies involving humans were approved by the Ethics Committee of Shanghai Pulmonary Hospital. The studies were conducted in accordance with the local legislation and institutional requirements. The participants provided their written informed consent to participate in this study.
